# Expression of RRBP1 in epithelial ovarian cancer and its clinical significance

**DOI:** 10.1042/BSR20190656

**Published:** 2019-07-23

**Authors:** Jing Ma, Sujing Ren, Jing Ding, Shuang Liu, Jiaqi Zhu, Rong Ma, Fanling Meng

**Affiliations:** Department of Gynecology, Harbin Medical University Cancer Hospital, Harbin, China

**Keywords:** Epithelial ovarian cancer, Immunohistochemistry, Prognosis, qRT-PCR, RRBP1, Western blotting

## Abstract

Hematopoietic pre-B cell leukemia transcription factor (PBX)-interacting protein (RRBP1) has been shown to participate in various aspects of malignancies. The clinical significance of RRBP1 and its involvement in the epithelial ovarian cancer have yet to be studied. The aim of the present study was to investigate the expression of RRBP1 in epithelial ovarian cancer (EOC) and its relationship with clinical characteristics and prognosis. We evaluated the mRNA and protein expression levels of RRBP1 by quantitative real-time polymerase chain reaction (qRT-PCR) and Western blotting (*n*=45). Immunohistochemistry and data analysis were used to examine the relationship between the expression level of RRBP1 and the clinicopathological features and prognosis of epithelial ovarian cancer. RRBP1 was highly expressed in EOC (*P*<0.001). The specimens were obtained from 108 patients undergoing surgery to treat epithelial ovarian cancer. RRBP1 expression was obviously related to Federation International of Gynecologie and Obstetrigue (FIGO) stage (*P*<0.001), histological grade (*P*=0.021), histological type (*P*=0.004), and lymph node metastasis (*P*=0.012) but was not related to patient age (*P*=0.385) or preoperative carbohydrate antigen125 (CA125) level (*P*=0.238). Univariate analysis showed that the prognosis of the epithelial ovarian cancer patients was related to the age of the patients, the FIGO stage, and the expression level of RRBP1 (*P*<0.05). Patients with higher RRBP1 expression had significantly worse overall survival (OS) (*P*=0.003) and disease-free survival (DFS) (*P*<0.001). Multivariate survival analysis proved that RRBP1 was an independent predictor of OS (*P*=0.003) and DFS (*P*<0.001). RRBP1 plays an important role in predicting the prognosis of EOC. These results show that RRBP1 is a potential target for the treatment of epithelial ovarian cancer.

## Introduction

Ovarian cancer is one of the most deadly gynecological tumors [1]. It is characterized by a lack of early clinical symptoms, and it is often detected at the last stage [2,3]. Although most patients initially respond to surgical debulking and chemotherapy, [4] the 5-year survival rate for ovarian cancer patients is only 20–40%; these low rates are mainly due to the occurrence of metastasis and recurrence [5]. Patients whose cancer is detected and treated in the early stages of the disease have significantly higher survival rates than those whose cancer is detected in the later stages; the survival rates for the former group can exceed 90% [6]. Therefore, further study on the identification of novel markers that can help detect tumor occurrence and metastasis is important for examining the development and treatment of ovarian cancer.

Hematopoietic pre-B cell leukemia transcription factor (PBX)-interacting protein (RRBP1) is an amino acid protein consisting of a hydrophobic NH2-terminus that includes a membrane-binding domain, a ribosome-binding domain, and an acidic coiled-coil COOH-terminal domain. RRBP1 is mainly located in the endoplasmic reticulum (ER), where it plays an important role in the intracellular transport of proteins in mammalian cells and participates in the secretion of proteins [7–10]. Studies in yeast have shown that RRBP1 also plays a role in ER stress and the associated unfolded protein response (UPR) [11,12]. In addition, RRBP1 stabilizes mRNA in yeast, but its mediated stabilization does not depend on the UPR [13]. It has been reported that RRBP1 also binds to the kinesis protein KFI5B, which is highly expressed in several cancer cell lines [14]. Recently, RRBP1 has been found to have a higher frequency of overexpression in colorectal cancer, [15,16] lung cancer, [17], and breast cancer [18,19]. Additionally, RRBP1 has become a potential marker of poor prognosis in colorectal cancer and breast cancer. Moreover, there are already data showing that RRBP1 plays an important role in the survival of tumor cells, the maintenance of malignant tumors and the adaptation of ER stress [15,20]. Therefore, it is very valuable to explore the impact of RRBP1 on the diagnosis and prognosis of ovarian cancer. Further verification of the possibility of RRBP1 as an independent tumor biomarker is also necessary for epithelial ovarian cancer.

In the present study, the clinicopathological effects of RRBP1 in epithelial ovarian cancer and their association with prognosis were analyzed and validated.

## Materials and methods

### Patients and treatment

In the present study, we examined 108 tumor specimens for immunohistochemistry. Specimens were obtained from patients with epithelial ovarian cancers at the Department of Gynecology of the the Harbin Medical University Cancer Hospital from February 2010 to June 2013. Tumor specimens were collected during surgery. None of the patients underwent relevant cancer treatment before surgery. The first operation of the patient aims to achieve maximum resection of the tumor, and a standard chemotherapy regimen of carboplatin plus paclitaxel is given postoperatively.

The patients’ ages ranged from 35 to 75, and the median age was 55 years. The follow-up period for patients was April 2018, the follow-up time was between 1 and 96 months, and the median follow-up time was 48 months. All clinical information to be studied is summarized and listed in Table 1. The normal samples in the present study were selected from 16 women undergoing hysterectomy for hysteromyoma at the Department of Gynecology of the Harbin Medical University Cancer Hospital.

**Table 1:** Association analyses between the expression levels of RRBP1 and the clinicopathological characteristics of epithelial ovarian cancer (EOC)

Variables	Patients	RRBP1	expression		*P*^1^
	*n*	Low	Moderate	High	
All cases					
Age (years)					
≤55	61	7	10	44	*P*=0.385
>55	47	4	4	39	
FIGO stage					
I	9	8	1	0	*P*<0.001
II	14	1	11	2	
III	80	2	2	76	
IV	5	0	0	5	
Histological grade					
G1	14	4	3	7	*P*=0.021
G2/G3	94	7	11	76	
Histological type					
Serous	91	5	10	76	*P*=0.004
Mucinous	7	2	2	3	
Endometrioid	6	2	1	3	
Clear cell	4	2	1	1	
CA125 (U.ml^−1^)					
≤35	7	2	1	4	*P*=0.238
>35	101	9	13	79	
Lymph node metastasis					
No	85	11	14	60	*P*=0.012
Yes	23	0	0	23	

Abbreviations: CA125, carbohydrate antigen125; EOC, epithelial ovarian cancer; FIGO, International Federation of Gynecology and Obstetrics; G1, well differentiated; G2, moderately differentiated; G3, poorly differentiated; RRBP1, Hematopoietic pre-B cell leukemia transcription factor (PBX)-interacting protein. ^1^χ^2^ test.

The institutional ethics committee approval for the project was obtained from the Medical Ethics Committee of The Affiliated Tumor Hospital of Harbin Medical University before the study was started, and informed consent was obtained from each patient.

### Western blot analysis

Forty-five samples were frozen in liquid nitrogen. The lysate was mixed proportionally with a protease inhibitor and used to cleave the extracted protein. The BCA method was then used to determine the protein concentration in the sample solution. The protein separated by 10% sodium dodecyl sulfate polyacrylamide gel electrophoresis was immediately transferred to a polyvinylidene fluoride membrane. After the transfer, a 5% skim milk powder solution was placed and sealed at room temperature. The primary antibodies against RRBP1 (1:500, Abcam, Anti-RRBP1 antibody ab95983) and β-actin (Santa Cruz Biotechnology, Santa Cruz, CA) were then diluted with a 5% skim milk powder solution according to the antibody instructions and incubated with the membrane overnight at 4°C. Similarly, the secondary antibody was incubated for 45 min, and the membrane was removed for exposure identification.

### Real-time PCR

The expression of RRBP1 mRNA was quantified by RT-PCR. Forty-five samples were used for real-time PCR, including twenty-nine epithelial ovarian cancer samples and sixteen normal tissue samples. Total RNA was isolated using an TRNzol reagent (BioTeke, Beijing, China). The concentration of RNA in each sample was determined using an ultraviolet spectrophotometer NANO 2000. The RNA sample obtained above was subjected to reverse transcription to obtain a corresponding cDNA. This step was done by the RCR instrument. Primers for RRBP1: Forward, 5′-AACCTAATGGGAAGATACCTGA-3′; Reverse, 5′-CATGGCTGGAACTGTGGC-3′. The primer sequence for β-actin as a reference is: Forward, 5′-CGGGAAATCGTGCGTGAC-3′; Reverse, 5′-GTCAGGCAGCTCGTAGCTCTT-3′. In this experiment, fluorescence quantitative analysis was performed using an ExicyclerTM 96 fluorescence meter (BIONEER, Korea). Relative RRBP1 abundances were determined with the 2^−ΔΔ*C*^_t_ method. This experiment was repeated three times.

### Immunohistochemical staining

Epithelial ovarian cancer tissue was cut into 4-micron-thick serial sections, fixed in formalin and embedded in paraffin. These paraffin sections were baked at 65°C for 30 min. To deparaffinize and rehydrate, the slides were placed in hydrogen peroxide (concentration 3%). All slides were immersed in 0.01 mol/l EDTA, autoclaved at 121°C for 4 min, cooled to room temperature, immersed three times with distilled water for 2 min, and washed three times with PBS for 5 min. Anti-RRBP1 antibody (Abcam, Anti-RRBP1 antibody ab95983) was used overnight at 4°C at a 1:400 dilution. The antibody was then immobilized three times with PBS for 5 min, placed into the moisture box, supplemented with the secondary antibody, and then placed in the humidor for 20 min at room temperature. The slides were counterstained with Hematoxylin, dehydrated, sealed, and placed in an oven at 60°C for 48 h.

### Staining assessment and scoring

The RRBP1 immunohistochemical expression score was scored using a semiquantitative method. Based on the number of positive tumor cells, the staining was scored as follows: level 0 (<10%), level 1 (10–33%), level 2 (34–66%), and level 3 (67–100%). The staining intensity was rated as 0–3 points according to negative, weak, moderate and strong staining. The final score is the sum of the two indicators.

In all samples, 0–1 was considered low expression, 2–3 was considered moderate expression, and >3 was considered high expression.

### Statistical analysis

The difference in RRBP1 expression between epithelial ovarian cancer tissues and normal tissues was evaluated by Western blotting and quantitative real-time polymerase chain reaction (qRT-PCR). The relationship between the level of RRBP1 expression and the different clinicopathologic factors was analyzed using the χ^2^ test. The correlation between the survival of patients with epithelial ovarian cancer and the level of RRBP1 expression was depicted and analyzed using the Kaplan–Meier method. At the same time, the impact of various clinical factors on the prognosis of epithelial ovarian cancer patients was analyzed by the log-rank test. A Cox regression model was performed for multivariate analysis of independent prognostic factors. Statistical significance was defined as *P*<0.05. All statistical analyses were carried out using the statistical software package SPSS V21.0.0.

## Results

### RRBP1 expression in patients with epithelial ovarian cancer

**Figure 1:**
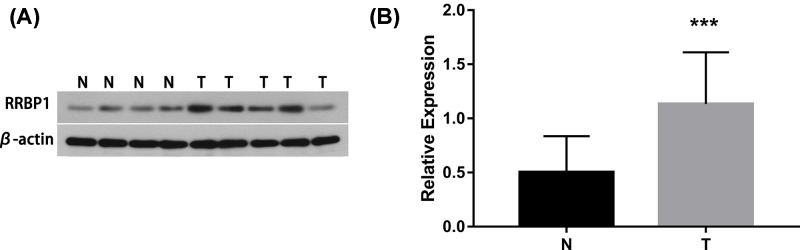
RRBP1 protein expresstion in EOCs by Western blot (**A**) Representative protein samples obtained from frozen normal epithelial ovarian tissues (N) and epithelial ovarian cancer tissues (T) were analyzed by Western blot. The levels of β-actin were used as an internal control; (**B**) Histogram of pooled data from N (*n*=16) and EOCs (*n*=29). RRBP1 expression was elevated in EOCs compared with that in N. The data are presented as the mean ± s. d. (^***^P<0.05).

The difference in RRBP1 expression between epithelial ovarian cancer tissues and normal tissues was evaluated by Western blotting experiments and Real-time PCR. Western blotting results showed the expression of RRBP1 was excessive at protein level in epithelial ovarian cancer samples (*P*<0.05, Figure 1). Real-time PCR results showed that the expression of RRBP1 at mRNA level in epithelial ovarian cancer tissues is much higher than that in normal epithelial ovarian cancer tissues (*P*<0.05, Figure 3).

### RRBP1 expression is associated with clinicopathological feature in epithelial ovarian cancer

**Figure 2:**
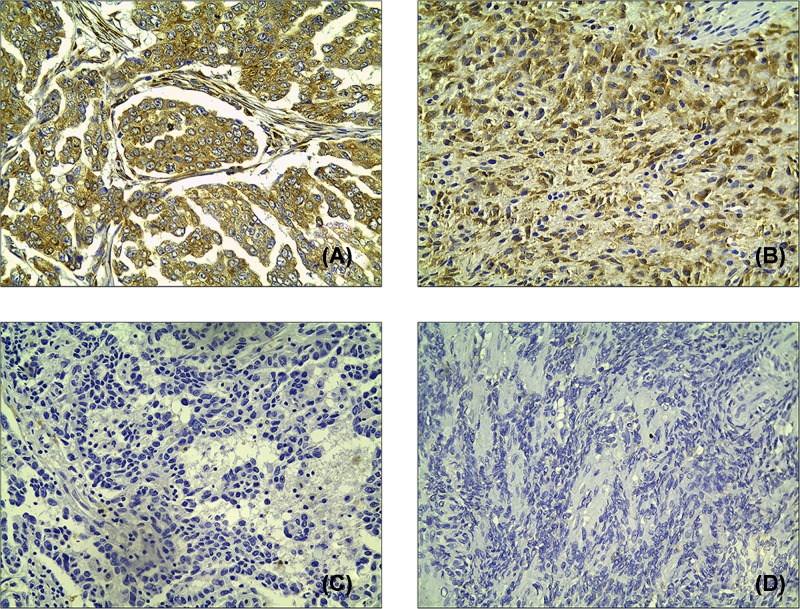
RRBP1 expression in EOCs by immunohistochemistry Immunohistochemical staining of RRBP1 in EOC specimens: (**A,B**) High expression of RRBP1 in EOCs; (**C,D**) Low expression of RRBP1 in EOCs.

The expression of RRBP1 in epithelial ovarian cancer tissues was analyzed by immunohistochemistry. Results of immunohistochemical staining are shown in Figure 2A–D. In the 108 epithelial ovarian cancer specimens, statistical analysis showed that 77% of cases had high expression, 13% of cases had moderate expression, and 10% had low expression. Moreover, the high RRBP1 expression in epithelial ovarian cancer was closely related to Federation International of Gynecologie and Obstetrigue (FIGO) stage (*P*<0.001), histological grade (*P*=0.021), histological type (*P*=0.004), and lymph node metastasis (*P*=0.012). High RRBP1 expression was not related to age (*P*=0.0385) or preoperative carbohydrate antigen125 (CA125) value (*P*=0.238). All statistical results are tabulated and summarized in Table 1.

**Figure 3:**
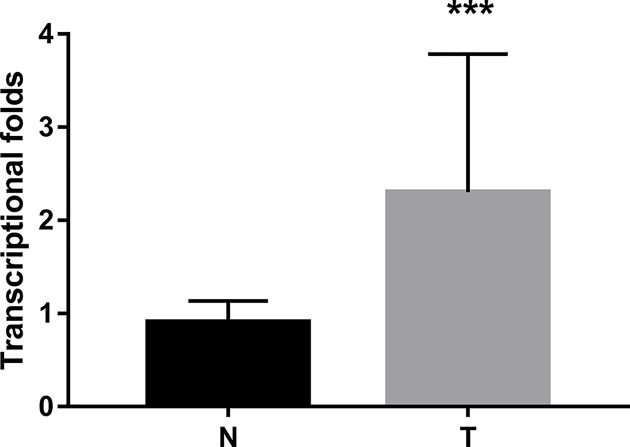
RRBP1 expression in EOCs by RT-PCR Histogram of RRBP1 mRNA expression in normal epithelial ovarian tissues and epithelial ovarian cancer tissues (N, normal epithelial ovarian tissues; T, epithelial ovarian cancer tissues). The levels of β-actin were used as an internal control, and the RRBP1 mRNA expression was calculated by 2^−∆∆*C*^_t_ method. RRBP1 mRNA expression was elevated in EOCs compared with normal cervical tissues. The data are presented as the mean ± s. d. (^***^P<0.05).

### The influence of RRBP1 expression on the survival of patients with epithelial ovarian cancer

**Figure 4:**
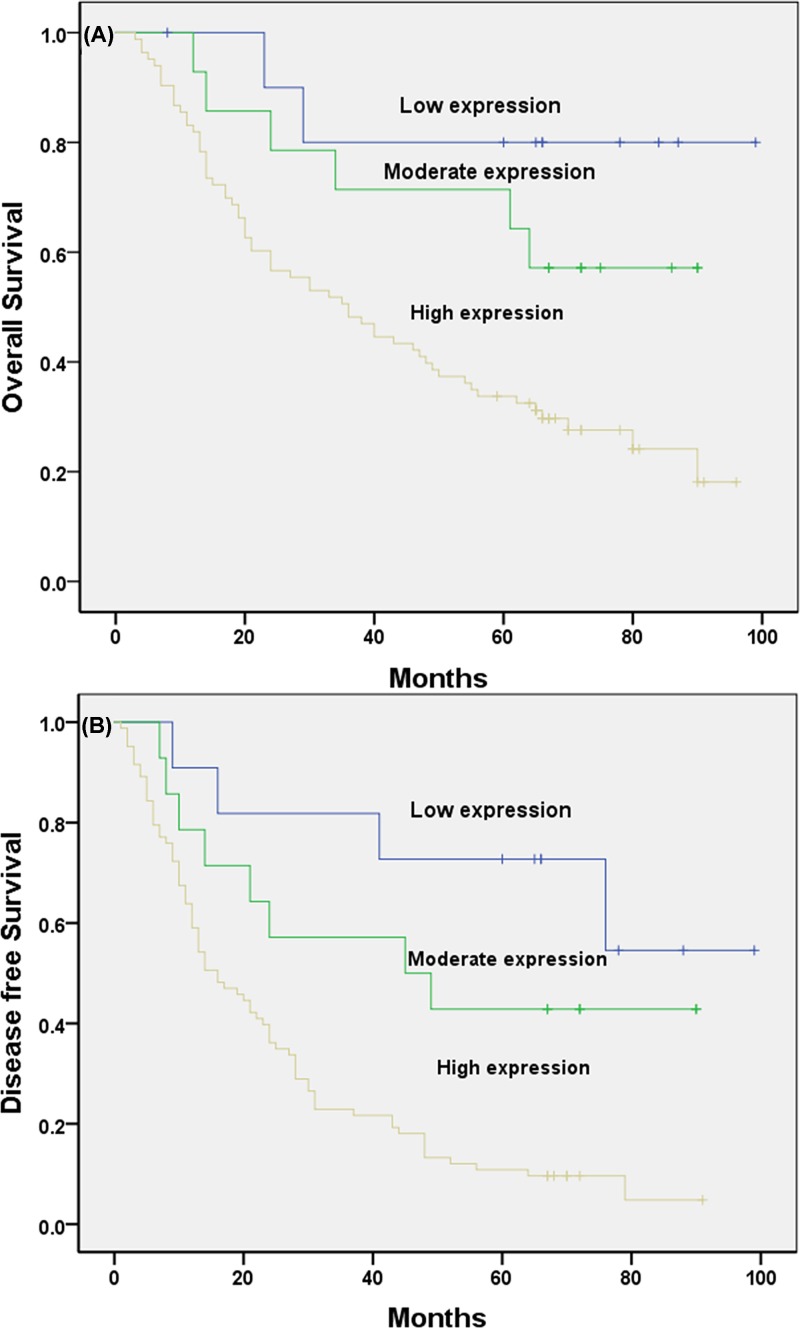
Kaplan–Meier analysis of OS and DFS related to the expression of RRBP1 Patients with high expression of RRBP1 had a poorer prognosis than those with low expression of RRBP1. (**A**) OS curves of the EOC according to their RRBP1 expression status, *P*=0.003; (**B**) DFS curves of the EOC patients according to their RRBP1 expression status, *P*<0.001.

Just as the Kaplan–Meier analysis shows, the high expression of RRBP1 significantly affects the overall survival (OS) or disease-free survival (DFS) of patients (Figure 4A,B; Table 2. *P*=0.003 and *P*<0.001, respectively). Moreover, the univariate survival analysis found that the patient age and the pathological type are also related to the prognosis of the patient (Table 2, all *P*<0.05).

**Table 2:** Univariate survival analysis of OS and DFS in 108 patients with EOC

Variables	*n*	OS		*P*^1^	DFS		*P*^1^
		Mean ± SE (month) 95% CI			Mean ± SE (month) 95% CI		
Age (years)							
≤55	61	60 ± 5	51−69	*P*=0.026	40 ± 5	31−49	*P*=0.045
>55	47	42 ± 5	42−52		25 ± 3	18−31	
FIGO stage							
I	9	82 ± 10	62−103	*P*=0.004	69 ± 12	46−92	*P*=0.001
II	14	75 ± 8	59−91		56 ± 9	38−73	
III	80	45 ± 4	36−52		27 ± 3	21−32	
IV	5	34 ± 11	13−56		26 ± 9	9−43	
Histological grade							
G1	14	64 ± 9	46−82	*P*=0.361	49 ± 9	31−68	*P*=0.127
G2/G3	94	50 ± 4	43−58		31 ± 3	25−37	
Histological type							
Serous	91	54 ± 4	46−61	*P*=0.358	34 ± 2	27−40	*P*=0.706
Mucinous	7	39 ± 14	11−67		33 ± 13	6−59	
Endometrioid	6	50 ± 15	21−80		34 ± 11	12−55	
Clear cell	4	31 ± 12	8−54		20 ± 10	1−39	
CA125 (U.ml^−1^)							
≤35	7	44 ± 11	23−65	*P*=0.654	31 ± 12	8−55	*P*=0.777
>35	101	53 ± 4	46−60		35 ± 3	28−41	
Lymph node metastasis							
No	85	53 ± 4	45−61	*P*=0.785	37 ± 4	30−45	*P*=0.118
Yes	23	50 ± 7	37−64		24 ± 4	16−33	
RRBP1							
Low expression	11	84 ± 9	66−103	*P*=0.003	74 ± 10	53−94	*P*=0.000
Moderate expression	14	66 ± 8	50−82		51 ± 9	33−70	
High expression	83	45 ± 4	38−52		25 ± 3	20−31	

Abbreviations: CI, confidence interval; FIGO, International Federation of Gynecology and Obstetrics; G1, well differentiated; G2, moderately differentiated; G3, poorly differentiated; RRBP1, Hematopoietic pre-B cell leukemia transcription factor (PBX)-interacting protein.^1^Log-rank test.

### Independence of RRBP1 expression from other clinicopathological factors

To assess whether the prognosis prediction ability of RRBP1 expression is independent of other clinicopathological features of patients with epithelial ovarian cancer, we performed multivariate Cox regression analysis. The data show that RRBP1 is an independent prognostic factor for both OS (Table 3, confidence interval [CI] = 1.346−4.102, *P*=0.003) and DFS (Table 3, CI = 1.554−3.709, *P*<0.001) in patients with epithelial ovarian cancer.

**Table 3:** Multivariate survival analysis of OS and DFS in 108 patients with EOC

Variables	OS			DFS		
	Exp (B)	95% CI	*P*^1^	Exp (B)	95% CI	*P*^1^
FIGO stage	1.408	0.718−2.761	*P*=0.320	0.971	0.538−1.753	*P*=0.922
RRBP1	2.350	1.346−4.102	*P*=0.003	2.397	1.554−3.790	*P*<0.001
Age	1.587	0.987−2.551	*P*=0.057	2.427	1.418−0.965	*P*=0.072

Abbreviations: FIGO, International Federation of Gynecology and Obstetrics; RRBP1, hematopoietic pre-B cell leukemia transcription factor (PBX)-interacting protein; CI, confidence interval.^1^Cox regression test.

## Discussion

In the present study, we examined the expression level of RRBP1 in epithelial ovarian cancer by immunohistochemical experiments involving 108 epithelial ovarian cancer samples. At the same time, we selected 29 cases of cancer tissue specimens and 16 normal tissue specimens for Western blotting and qRT-PCR to study the expression of RRBP1 at the protein and mRNA levels. The Western blotting and qRT-PCR results confirmed that the expression of RRBP1 in epithelial ovarian cancer tissues was much higher than that in normal tissues. Overexpression of RRBP1 in epithelial ovarian cancer was shown to be obviously related to FIGO stage, histological grade, histological type, and lymph node metastasis. In addition, our data showed that epithelial ovarian cancer patients with RRBP1 overexpression had significantly decreased OS and DFS. RRBP1 is an independent prognostic factor for epithelial ovarian cancer. These findings indicate that RRBP1 plays an important role in the progression of epithelial ovarian cancer and that it has a profound effect on the prognosis of epithelial ovarian cancer. To our knowledge, this is the first examination to demonstrate the overexpression of RRBP1 in epithelial ovarian cancer and to suggest that RRBP1 may be a valuable prognostic biomarker for epithelial ovarian cancer.

Earlier research reports on RRBP1 in tumors, including breast cancer, [[18,19] lung cancer, [17] and colorectal cancer, [15,16] have found that RRBP1 plays an important role in the prognosis of tumors. The role of RRBP1 in epithelial ovarian cancer was consistent with the results of the current study and proved the connection between the overexpression of RRBP1 in epithelial ovarian cancer and adverse biological behavior. These findings suggest that RRBP1 plays an important biological role in the development of cancer and tumor progression. The above results improve the possibility that RRBP1 may be a prognostic parameter of ovarian cancer, suggesting that RRBP1 can be used for individualized treatment and prognostic evaluation in patients with epithelial ovarian cancer.

Many studies have explained the mechanism by which RRBP1 promotes the development of cancer. IMT study by Lee et al. [21] provides the first proof of the mechanism of recurrent carcinogenesis in RRBP1-ALK. The results of the study by Nazarian et al. [10] showed that the expression of RRBP1 regulates RNA stability by localizing the ER membrane at the mRNA conversion level rather than at the transcriptional level, which may be achieved by locating mRNA on the ER membrane and protecting it from cell cycle-dependent degradation. Tsai et al. [17] found that the carcinogenic mechanism of RRBP1 is related to the UPR pathway and that RRBP1 may attenuate ER stress and help tumors survive tumorigenesis. Their findings also suggested that RRBP1 may participate in the accumulation of perinuclear autophagosomes in cancer cells by interacting with the kinesin family member 5B (KIF5B) [16]. During tumorigenesis, adaptive stress responses cause UPRs and initiate the activation of survival cascade mechanisms. In some reports, the increase in RRBP1 protein expression in tumor cells is attributed to increased RRBP1 mRNA due to gene amplification and/or elevated transcription [16,18]. Gao et al. [22] studied the regulation of RRBP1 at the translation level and found that La autoantigen induced RRBP1 expression under cellular stress through the internal ribosomal entry site (IRES). The current research report further proves that RRBP1 participates in and promotes the occurrence and development of cancer, but the specific mechanism of action has yet to be elucidated and warrants further research.

The significance of RRBP1 cancer research is to use the gene as a potential target for cancer therapy. Research results have shown that RRBP1 knockdown can affect the stability of ATF6 and GRP78 mRNA, thereby reducing the *in vivo* tumorigenicity of lung cancer cells [17]. Studies have shown that UTR-induced trastuzumab resistance may be a cause of poor prognosis in patients with RRBP1 expression [19]. The above studies indicate the possibility of RRBP1 as a new idea for curing epithelial ovarian cancer. Of course, basic research needs further support.

## Conclusions

In summary, RRBP1 is overexpression in most epithelial ovarian cancer patients, and overexpression of RRBP1 in epithelial ovarian cancer leads to an even worse prognosis in patients with epithelial ovarian cancer. However, to effectively apply the effect of RRBP1 in tumors to the curing of tumors, it needs to do more accurate experiments are needed to verify this hypothesis.

## Availability of data and materials

The data used and analyzed during the current study are available from the corresponding author on reasonable request.

## Abbreviations

CA125: carbohydrate antigen125
CI: confidence interval
DFS: disease-free survival
EOC: epithelial ovarian cancer
ER: endoplasmic reticulum
FIGO: Federation International of Gynecologie and Obstetrigue
OS: overall survival
qRT-PCR: quantitative real-time polymerase chain reaction
RRBP1: Hematopoietic pre-B cell leukemia transcription factor (PBX)-interacting protein
UPR: unfolded protein response
